# Soluble Forms of the Receptor for Advanced Glycation Endproducts (RAGE) in Periodontitis

**DOI:** 10.1038/s41598-019-44608-2

**Published:** 2019-06-03

**Authors:** Laurent Detzen, Bin Cheng, Ching-Yuan Chen, Panos N. Papapanou, Evanthia Lalla

**Affiliations:** 1 Division of Periodontics, College of Dental Medicine, Columbia University, New York, NY USA; 2 Department of Periodontology, Service of Odontology, Rothschild Hospital, AP-HP & UFR of Odontology, Paris 7-Denis Diderot University, Paris, France; 3 Department of Biostatistics, Mailman School of Public Health, Columbia University, New York, NY USA

**Keywords:** Chronic inflammation, Dental diseases

## Abstract

The receptor for advanced glycation endproducts (RAGE) is critically involved in the pathobiology of chronic inflammatory diseases. Soluble forms of RAGE have been proposed as biomarkers of severity in inflammatory and metabolic conditions, and in monitoring therapeutic responses. The aim of the present study was to determine circulating levels of the soluble forms of RAGE in periodontitis and to evaluate the expression of cell-bound, full-length RAGE and its antagonist AGER1 locally, in gingival tissues. Periodontitis patients and periodontally healthy, sex- and age-matched controls (50 per group) were included. Serum levels of total soluble RAGE and cleaved RAGE (cRAGE) were significantly lower in periodontitis patients. Levels of the endogenous secretory esRAGE were similar in the two groups. cRAGE remained significantly lower in the periodontitis group following multiple adjustments, and had a statistically significant inverse correlation with body mass index and all periodontal parameters. In periodontitis patients, gene expression of full-length RAGE and of AGER1 were significantly higher in periodontitis-affected gingival tissues compared to healthy gingiva. Soluble forms of RAGE, particularly cRAGE, may serve as biomarkers for the presence and severity/extent of periodontitis, and may be implicated in its pathogenesis and its role as a systemic inflammatory stressor.

## Introduction

The Receptor for Advanced Glycation Endproducts (RAGE) is a transmembrane protein which belongs to the immunoglobulin superfamily^1^. RAGE was originally identified for its ability to bind to Advanced Glycation Endproducts (AGEs) and was subsequently found to be a pattern recognition receptor able to recognize a wide class of ligands, including high mobility group box-1 (HMGB1)/amphoterin, S100/calgranulins, and amyloid-β peptide^2–5^. Ligand engagement and RAGE’s transduction upregulate various pro-inflammatory mediators and adhesion molecules, contributing to activation of many cellular processes such as proliferation, apoptosis, autophagy, migration, and notably inflammation and immune response^6^. Moreover, RAGE mediates its pathological actions by the generation of oxidative stress through the activation of NADPH oxidase and the transcription factor NF-κB^7,8^. RAGE is highly expressed during embryogenesis, but kept at low levels in adults. In environments of cellular stress, such as inflammation, RAGE is overexpressed^9,10^. The RAGE-ligand axis is intimately involved in the pathophysiology and complications of a wide range of chronic conditions such as diabetes mellitus, vascular disease, arthritis, aging, cancer, and neuro-degeneration^6,11–14^. In addition, the RAGE-AGE axis is one of the key pathogenic mechanisms involved in the periodontal destruction associated with diabetes^15^.

Soluble forms of RAGE (sRAGE) exist, and there are at least two mechanisms by which they may be generated^16^. First, total extracellular RAGE is cleaved from the cell surface by the action of matrix metalloproteinases (MMPs) and A-disintegrin and metalloprotease (ADAM)-10; this form is known as cleaved RAGE (cRAGE)^17,18^. Second, alternative mRNA splicing results in multiple variants among which endogenous secretory RAGE (esRAGE), also termed RAGE_v1, is the primary secreted, soluble isoform^19,20^. Importantly, esRAGE contains a unique sequence of amino acids in its C2-Ig domain that has been used to generate a specific antibody (also available in an ELISA kit) that detects only this protein and does not cross-react with other forms of sRAGE.

Soluble forms of RAGE have been gauged in the circulation and in other body fluids, and several studies have considered them as biomarkers of interest in evaluating disease severity and in monitoring patient response to therapy^21,22^. They act as decoy receptors, inhibiting ligand-RAGE interactions, and have generally been described as protective forms, mitigating the deleterious effects of the activation of the full-length receptor^23,24^. Indeed, expression of sRAGE has been shown to be decreased in pathologies such as atherosclerosis, coronary artery disease, essential hypertension, rheumatoid arthritis and Alzheimer’s disease in individuals without diabetes^25–27^. In periodontitis, a chronic, microbially-induced inflammatory disorder that affects the structures that support teeth and can adversely affect general health^28^, we have previously shown that the administration of exogenous sRAGE blocks the activation of full-length RAGE, reducing alveolar bone destruction and gingival inflammation in diabetic mice^29^.

Another transmembrane protein, Advanced Glycation Endproduct Receptor 1 (AGER1), mediates the uptake, degradation and disposal of AGEs and has been involved in cell survival and regulation of reactive oxygen species^30^. AGER1 is encoded by the gene *DDOST* and demonstrates an antagonistic effect to full-length RAGE, suppressing the heightened inflammation and oxidative stress generated by RAGE activation. The AGER1 to RAGE ratio has thus been proposed as an important element of the defense against excessive oxidative stress^31^. The potential role of the balance between RAGE and AGER1 has not been explored in the context of periodontitis.

Therefore, the aim of the present study was to determine the circulating levels of the different soluble forms of RAGE in periodontitis, and to evaluate the expression of cell-bound RAGE and its antagonist AGER1 locally, in gingival tissues.

## Results

### Study population

The study participants consisted of 50 periodontitis patients and 50 periodontally healthy, sex- and age-matched controls. By design, all periodontitis-related parameters examined - percent of sites with dental plaque, percent of periodontal pockets with bleeding on probing, mean pocket depth, percent of deep periodontal pockets (≥5 mm), and mean attachment loss - were significantly different between the two groups. Also by design, the sex distribution was the same (50% men in each group) as was the age distribution (mean age was 42.9 ± 9.9 years in the periodontitis group and 43.0 ± 9.8 years in the control group). Body Mass Index (BMI) was found to be significantly higher in periodontitis patients compared to periodontally healthy controls (27.9 ± 5.4 kg/m^2^ *vs*. 25.3 ± 4.7 kg/m^2^, respectively; two-sample t-test, p = 0.008).

### Serum levels of soluble forms of RAGE

**Figure 1:**
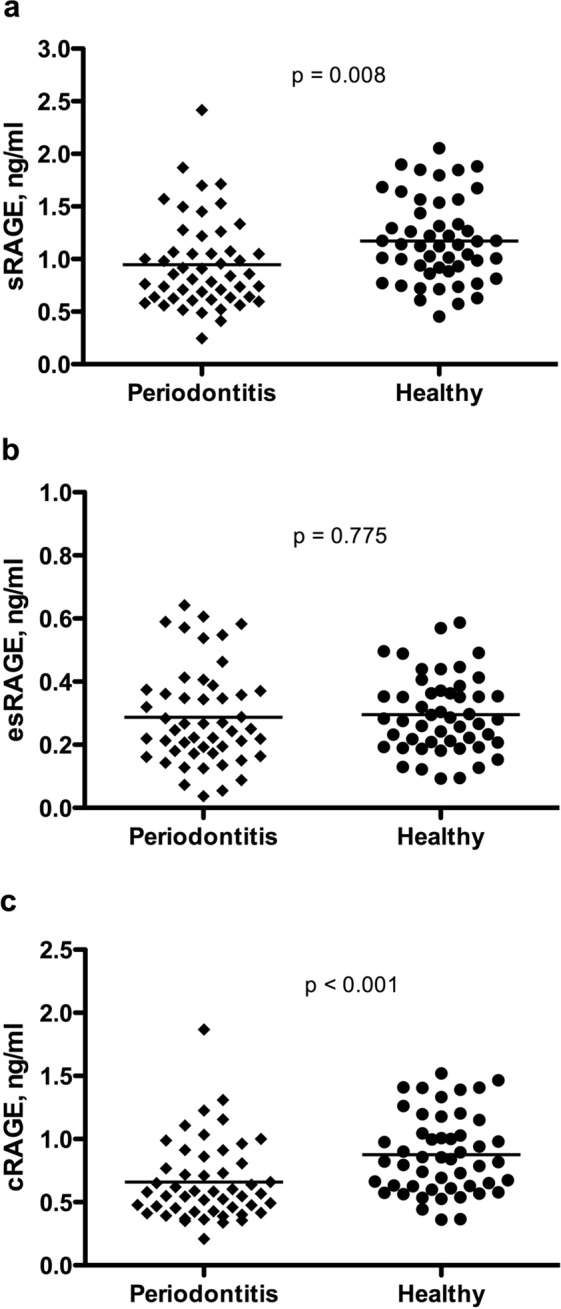
Serum levels of total soluble RAGE (**a**), endogenous secretory RAGE (**b**), and cleaved RAGE (**c**) in periodontitis patients and healthy controls (n = 50 per group). Horizontal lines represent mean values, p values are based on two-sample t tests.

As shown in Fig. 1, total sRAGE was significantly lower in the serum of periodontitis patients compared to periodontally healthy controls (0.95 ± 0.43 ng/mL *vs*. 1.17 ± 0.40 ng/mL, respectively; two-sample t-test, p = 0.008). Serum levels of esRAGE were similar in the two groups (periodontitis: 0.29 ± 0.15 ng/mL, control: 0.30 ± 0.12 ng/mL; two-sample t-test, p = 0.775). Levels of serum cRAGE were calculated (sRAGE - esRAGE) and found to be significantly lower in the periodontitis group compared to the control group (0.66 ± 0.31 ng/mL *vs*. 0.88 ± 0.79 ng/mL, respectively; two-sample t-test, p < 0.001).

The difference in cRAGE levels between periodontitis and periodontally healthy participants was statistically significant even after adjusting for age, sex and BMI in multiple linear regression analysis (t-test, p = 0.011). The difference in total sRAGE did not remain statistically significant following adjustments for age, sex and BMI (t-test, p = 0.085).

cRAGE represented the majority of total sRAGE in the study population and accounted for 70% of the total in the periodontitis group and 75% in the control group. When we examined quartiles of cRAGE level across the total study population (Table 1), we found that BMI and all periodontal parameters were statistically significantly different across the groups. Total sRAGE and esRAGE were also statistically significantly different among the 4 quartile groups. Results were similar when the population was stratified by total sRAGE quartiles.

**Table 1:** Study population stratified by quartiles of cleaved RAGE (cRAGE) serum level.

Variable	All (n = 100)	Q1 (n = 25)	Q2 (n = 25)	Q3 (n = 25)	Q4 (n = 25)	*p*
Age, years	43.0 ± 9.8	45.0 ± 8.5	40.8 ± 10.6	41.9 ± 8.2	44.1 ± 11.6	0.391
Sex, male	50 (50)	13 (52)	10 (40)	12 (48)	15 (60)	0.556
BMI, kg/m^2^	26.6 ± 5.0	30.5 ± 6.1	25.9 ± 4.1	25.4 ± 4.2	24.7 ± 3.2	**<0.001**
Number of teeth	26.1 ± 2.3	25.3 ± 2.6	26.8 ± 1.8	26.2 ± 2.2	26.1 ± 2.5	0.168
% sites with dental plaque	43.9 ± 26.7	61.6 ± 19.7	43.2 ± 27.1	37.4 ± 26.6	33.5 ± 25.1	**<0.001**
% bleeding pockets	37.7 ± 26.6	53.5 ± 22.0	38.5 ± 26.4	31.3 ± 28.5	27.6 ± 22.6	**0.002**
Mean pocket depth, mm	3.0 ± 1.0	3.6 ± 1.0	2.9 ± 1.1	2.9 ± 1.0	2.6 ± 0.8	**0.007**
% deep pockets (≥5 mm)	16.3 ± 20.3	28.5 ± 21.0	15.0 ± 20.3	13.4 ± 19.6	8.4 ± 15.5	**0.003**
Mean attachment loss, mm	2.3 ± 1.6	3.3 ± 1.6	2.1 ± 1.7	1.9 ± 1.4	1.8 ± 1.2	**0.001**
sRAGE, ng/ml	0.77 ± 0.33	0.63 ± 0.18	0.83 ± 0.11	1.13 ± 0.13	1.64 ± 0.28	**<0.001**
esRAGE, ng/ml	0.29 ± 0.14	0.21 ± 0.14	0.23 ± 0.09	0.30 ± 0.10	0.41 ± 0.13	**<0.001**

Data shown as mean ± SD or count (percent) for continuous and categorical variables, respectively. P values derived from two-sample t tests, except for sex where Chi-square test was used; significant p values are highlighted in bold.

### Correlations

**Figure 2:**
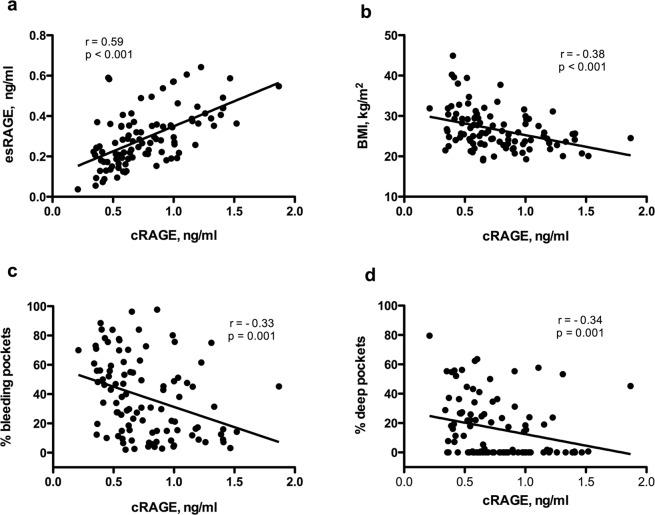
Correlation between serum levels of cleaved RAGE (cRAGE) and: (**a**) serum levels of endogenous secretory RAGE (esRAGE), (**b**) body mass index (BMI), (**c**) percent of periodontal pockets with bleeding on probing, and (**d**) percent of deep periodontal pockets (with probing depth ≥5 mm) in study participants. r: Pearson correlation coefficient; p values derived from Fisher’s z test; n = 100 for all.

Serum cRAGE was negatively and significantly correlated with BMI (*r* = −0.38, Fisher’s z test, *p* < 0.001) and all periodontal parameters measured. Its correlation with esRAGE was positive and significant (*r* = 0.59, Fisher’s z test, *p* < 0.001), but it was not significant with age (*r* = −0.03, Fisher’s z test, *p* = 0.782) or the number of teeth present (*r* = 0.08, Fisher’s z test, *p* = 0.405). Fig. 2 scatterplots show the correlation between cRAGE and esRAGE, BMI, percent of bleeding pockets and percent of deep pockets for all study participants (n = 100). Similarly to cRAGE, serum levels of total sRAGE were negatively and significantly correlated with BMI (*r* = −0.41, Fisher’s z test, *p* < 0.001) and all periodontal parameters.

### RAGE and AGER1 in gingival tissue

Gene expression of the full-length, cell-bound RAGE was assessed in gingival tissues from 20 periodontitis patients and was found to be significantly increased in periodontitis-affected sites compared to unaffected sites within the same patient (mean fold change in expression: 4.5 ± 7.1, t-test, p = 0.010). AGER1 gene expression was also significantly increased in periodontitis-affected gingival tissues (mean fold change compared to periodontally healthy tissues: 2.3 ± 3.5, t-test, p < 0.001).

**Figure 3:**
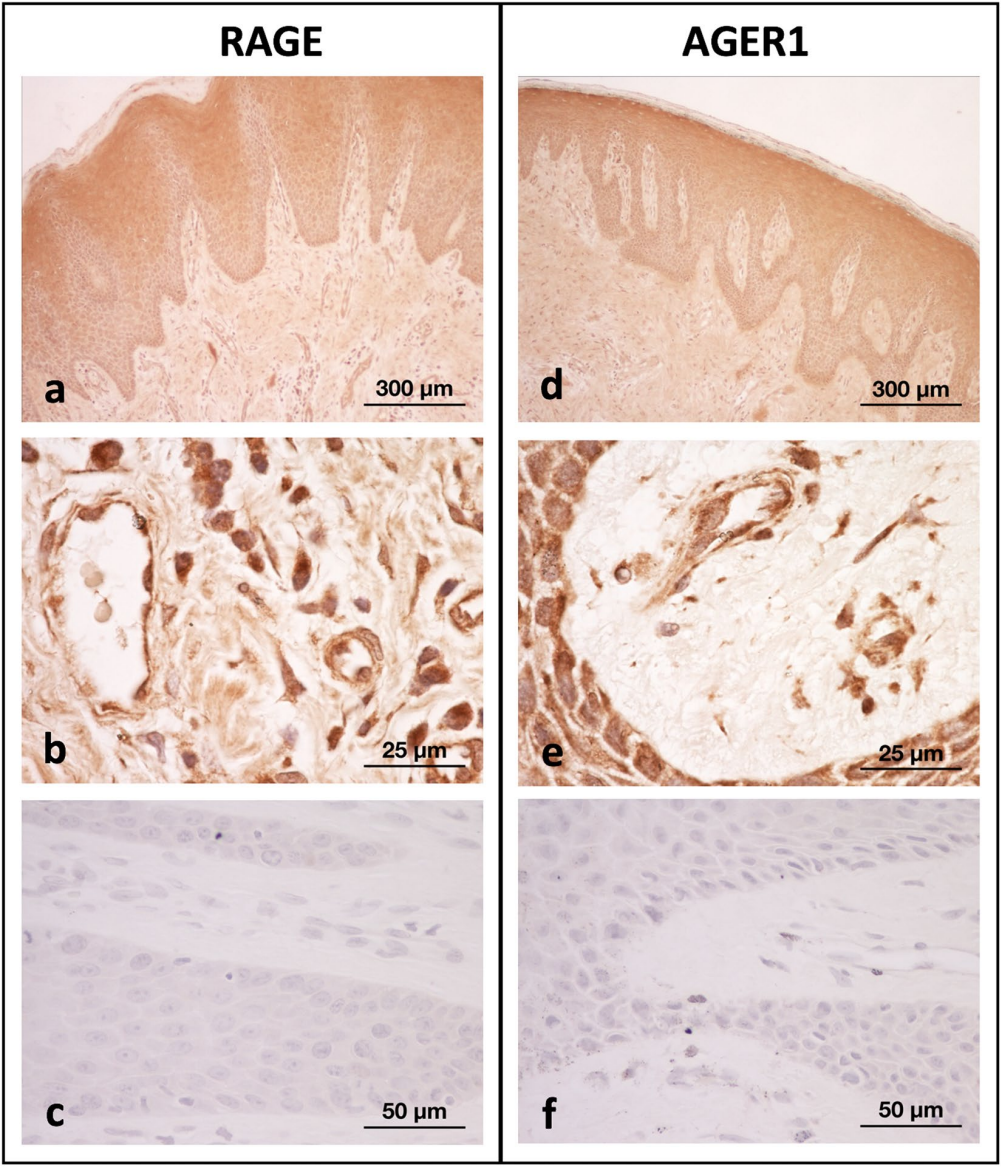
Immunohistochemistry of gingival tissue from periodontitis-affected sites for full-length RAGE (**a**,**b**) and AGER-1 (**d**,**e**); panels **c** and **f** show non-immune controls. Expression is evident in the epithelium; in the connective tissue, endothelial and inflammatory cells are positively stained for both proteins.

Immunostaining of gingival tissue from periodontitis-affected sites for both RAGE and AGER-1 proteins (Fig. 3) showed expression in epithelial cells. In the connective tissue, endothelial and inflammatory cells were positively stained for both proteins.

## Discussion

Our study demonstrates for the first time that circulating levels of total sRAGE are significantly lower in patients with periodontitis compared to age- and sex-matched periodontally healthy controls. Interestingly, levels of the endogenous secretory form esRAGE did not show any differences between the groups, but cleaved, cRAGE levels were significantly attenuated in the periodontitis group compared to the control group and remained significantly lower even after multiple linear regression analysis. cRAGE thus appears to be the soluble RAGE form of interest in periodontitis.

sRAGE has been shown to act as a decoy receptor, competitively inhibiting cell-bound RAGE activation and its deleterious effects. Moreover, soluble forms of RAGE lack the signaling ability of the full-length, cell-bound RAGE which critically affects cellular phenotype and function, leading to downstream pro-inflammatory and pro-oxidant effects. Decreased circulating levels of sRAGE and cRAGE in periodontitis patients in the present study suggest low interference with the ligand-RAGE axis which is known to lead to increased periodontal inflammation/destruction. This finding is also consistent with human data in other inflammatory, autoimmune or infectious diseases^22,32^.

BMI and waist/hip ratio have been previously correlated negatively with sRAGE in healthy subjects^33,34^. Similarly, in our study, both total sRAGE and cRAGE were negatively and significantly correlated with BMI. Further, the interplay between sex and the RAGE axis has been highlighted in previous studies, and sex and hormonal status in females have been suggested to influence levels of sRAGE and RAGE ligands^35,36^. In our study, we had equal numbers of men and women in each of the two groups. Although no information on the pre/post-menopausal status of female participants was available, mean age of women in the periodontitis group was 43.3 ± 9.8 years and mean age of women in the healthy group was 43.0 ± 10.0 years, suggesting homogeneity in this perspective as well.

Full-length RAGE has been shown to be significantly overexpressed in gingival tissues in patients with severe periodontitis, with or without diabetes, compared to those with healthy periodontium^37–39^. In the present study, to further dissect the involvement of cell-bound RAGE and, for the first time, of AGER1 in periodontitis we did not compare periodontitis *vs*. non-periodontitis subjects. Rather, and to avoid subject-based confounding factors, we assessed RAGE and AGER1 mRNA levels by qPCR in paired gingival tissue biopsies (periodontitis-affected vs. periodontally healthy gingiva) among periodontitis patients. Our analysis revealed a statistically significant, 4.5-fold increase in the expression of cell-bound RAGE in periodontitis-affected sites. Interestingly, AGER1 expression was also significantly increased, by 2.3-fold, in periodontitis-affected sites. Gingival tissue immunostaining showed that the same types of cells expressed both proteins, notably, endothelial and inflammatory cells in the connective tissue. In diabetes, RAGE activation has been shown to be exacerbated partly because of the inhibition of AGER1 expression under significant and long-lasting exposure to AGEs and oxidative stress^30^. In the absence of diabetes, but in the context of chronic periodontal inflammation, AGER1 is potentially upregulated to compensate for the increased expression of RAGE, but appears to only partially inhibit its activation.

Limitations of our study include the cross-sectional design and relatively small sample size. Our ability to detect sRAGE and esRAGE levels was dictated by the sensitivity of the assays used, and since there are no assays currently available to measure cRAGE, cRAGE levels were calculated rather than measured directly. Larger, longitudinal studies will shed more light into these novel observations.

Whether periodontal intervention has the potential to impact soluble isoforms of RAGE in the serum, or the expression of cell-bound RAGE and its antagonist AGER1 in tissues remains to be seen. Generally, in non-diabetic individuals, otherwise beneficial interventions appear to raise levels of sRAGE^16^.

Taken together, the data presented here suggest that soluble forms of RAGE may have a role in the pathogenesis of periodontitis and its widely-reported association with other systemic conditions. Specifically, circulating sRAGE, and particularly its cleaved form cRAGE, may be considered protective against periodontitis and the systemic inflammatory stress it can impose. The findings set the stage for future studies on the use of soluble forms of RAGE as valuable biomarkers of oral and/or systemic inflammation, and highlight that periodontitis can be a confounding factor when evaluating circulating levels of sRAGE in other disease states or following therapeutic interventions.

## Methods

### Recruitment of study participants and clinical examination

The study was approved by the Columbia University Institutional Review Board and in accordance with the Declaration of Helsinki, as revised in 2013. Participants were recruited among individuals attending the Columbia University College of Dental Medicine clinics in New York City and signed informed consent prior to enrollment. The inclusion criteria were age ≥21 years and at least 20 teeth present. Current smokers, individuals who quit smoking less that 6 months prior to recruitment, had used antibiotics, aspirin, or anticoagulants in the preceding 3 months, pregnant women, and individuals with diabetes mellitus, history of coronary heart disease, inflammatory bowel disease, rheumatoid arthritis, or malignancies were excluded from the study.

A full-mouth periodontal examination (six surfaces/sites per tooth at all teeth present, excluding 3^rd^ molars) was performed, using a manual periodontal probe (UNC-15; Hu-Friedy, Chicago, IL, USA). Probing depth and clinical attachment loss (assessing the severity of periodontal destruction) were measured and rounded to the nearest millimeter. Bleeding on probing (a sign of inflammation at the base of the periodontal pocket) and dental plaque (the bacterial biofilm that triggers periodontal inflammation) were recorded dichotomously (presence/absence). Height and weight were also recorded for all participants for determination of body mass index (BMI).

Patients assigned in the periodontitis group had at least two teeth in each quadrant with a probing depth ≥5 mm and concomitant attachment loss ≥3 mm, and bleeding on probing at ≥30% of their tooth surfaces. Periodontally healthy participants were sex- and age-matched, had no sites with probing depth ≥5 mm or interproximal attachment loss ≥3 mm.

### Blood sampling and assessment of sRAGE isoforms

A sample of 35 ml of venous blood was collected by venipuncture from each study participant using serum gel blood collection tubes (BD Vacutainer, Becton, Dickinson and Company, Franklin Lakes, NJ, USA). Blood samples were centrifuged at 1,300 *g* for 10 min, serum was collected, aliquoted, and stored at −80 °C. Serum levels of total sRAGE and of esRAGE were assessed by ELISA (R&D Systems, Minneapolis, MN, USA and B-Bridge International, Mountain View, CA, USA, respectively) according to the manufacturers’ protocols. There are no assays currently available to directly measure the concentration of cRAGE in biological fluids and, thus, cRAGE levels were calculated by subtracting the level of esRAGE from that of total sRAGE, as previously described^40–42^.

### Gingival tissue sampling

Two gingival tissue biopsies were harvested per subject from 20 patients with periodontitis. Periodontitis is site specific, and the first tissue sample was collected from a periodontitis-affected site, characterized by a pocket depth ≥4 mm, attachment loss ≥3 mm and positive for bleeding on probing. The second sample was collected from a periodontally healthy, control site, with a pocket depth ≤3 mm, attachment loss ≤2 mm and no bleeding on probing. Tissue samples were stored in liquid RNA stabilization reagent (RNAlater, Ambion, Austin, TX, USA) and then in liquid nitrogen until analysis.

### RAGE and AGER1 mRNA expression assessment

Specimens were homogenized in Trizol (Invitrogen Life Technologies, Carlsbad, CA, USA). After incubation with chloroform and centrifugation at 1,200 *g*, the upper aqueous phase was collected and RNA was precipitated by mixing with isopropyl-alcohol, followed by additional centrifugation and wash in 75% ethanol. The extracted RNA was purified using a total RNA isolation kit (RNeasy; Qiagen, Valencia, CA, USA) and quantitated spectrophotometrically. Reverse transcription was performed (Hight Capacity cDNA reverse transcription kit, Foster City, CA, USA). Published RAGE and AGER1 primers were selected (Primer Bank, https://pga.mgh.harvard.edu/primerbank/index.html) for Real Time Polymerase Chain Reaction (RT-PCR), and beta-actin was chosen as a housekeeping gene. Amplification reactions were prepared with SYBR Green (Power SYBR Green PCR Mastermix, Applied Biosystems) and performed using Step One Plus RT-PCR system (Applied Biosystems). Relative mRNA levels of target genes were determined by the 2^−ΔΔct^ method.

### Immunohistochemistry

Tissue sections were stained with haematoxylin and eosin solutions, mounted and examined. Deparaffinized and rehydrated sections were used for RAGE and AGER1 qualitative assessment using an indirect immunoperoxidase technique. Endogenous peroxidase and serum bovine serum albumin blocks were performed to reduce nonspecific reactions. Sections were incubated with primary monoclonal anti-RAGE antibody (R&D Systems) and anti-AGER1 antibody (Santa Cruz Biotechnology, Santa Cruz, CA, USA). After washing in PBS and secondary antibody incubation, sections were treated with the Avidin-Biotin complex (Vectastain Universal Elite ABC kit, Vector Laboratories, Burlingame, CA, USA) according to the manufacturer’s protocol. Negative/non-immune controls were obtained by omitting the primary antibody. The substrate 3,30-diaminobenzidine (DAB) was applied for the same amount of time on each section and, finally, sections were counter-stained with Mayer’s haematoxylin (Sigma, St. Louis, MO, USA) to visualize tissue topography.

### Data and statistical analyses

Probing depth and attachment loss were calculated as average of all sites for each subject. In addition, plaque, bleeding on probing and deep probing depth (≥5 mm) were calculated as percentage of all sites for each study participant. Continuous variables were compared between periodontitis and healthy subjects, and among the 4 quartile groups of cRAGE level, using independent two-sample t tests, and categorical variables were compared using the Chi-square test. Multiple linear regression and t-test were used to assess the impact of periodontal status (periodontitis/healthy) on serum sRAGE and cRAGE levels, controlling for age, sex, and BMI. The association between soluble forms of RAGE and BMI, age, number of teeth and periodontal parameters was assessed via Pearson’s correlation, and its significance was assessed by the Fisher’s z test. All statistical analyses were performed using SAS, version 9.4. A *p*-value < 0.05 was considered statistically significant. Data are presented as mean ± SD or count (percent) for continuous and categorical variables, respectively.

## Data Availability

The datasets generated and/or analyzed during the current study are available from the corresponding author on reasonable request.
